# Early Tracheostomy in Trauma Patients with Acute Respiratory Distress Syndrome due to Novel Coronavirus Disease 2019 (COVID-19)

**DOI:** 10.30476/BEAT.2020.86487

**Published:** 2020-07

**Authors:** Fariborz Ghaffarpasand, Mohammad Reza Saki, Nazanin Dadashpour, Zahra Ghahramani, Shahram Paydar

**Affiliations:** 1 Research Center for Neuromodulation and Pain, Shiraz University of Medical Sciences, Shiraz, Iran.; 2 Trauma Research Center, Rajaee (Emtiaz) Trauma Hospital, Shiraz University of Medical Sciences, Shiraz, Iran; 3 Department of Internal Medicine, Shiraz University of Medical Sciences, Shiraz, Iran

The novel coronavirus disease 2019 (COVID-19) caused by severe acute respiratory syndrome coronavirus 2 (SARS‑CoV‑2) was first reported in Wuhan, China in December 2019 [1, 2] and was rapidly spread all over the world, being announced as a pandemic on March 11^th^, 2020 by the World Health Organization (WHO) [3]. Approaching the mid of August, approximately 20 million people are infected worldwide and 720,000 have died due to the infection and its complications [4]. The virus causes respiratory infection and involves both the upper and lower respiratory tract as well as the gastrointestinal tract, hepatic, neurologic and renal systems [5]. COVID-19 disease ranges from mild to severe respiratory illness and could be asymptomatic in some individuals while causing respiratory failure requiring mechanical ventilation [6]. The treatment of the patients with acute respiratory distress syndrome (ARDS) due to COVID-19 is still to be identified and the current approaches and guidelines recommend supportive care along with mechanical ventilation [7]. 

Tracheostomy in patients with COVID-19 infection is a matter of research in the recent months and there is a great deal of controversy regarding the timing, method and candidates [8]. As the prolonged intubation is the most common indication of tracheostomy in the world and the COVID-19 patients require prolonged intubations, thus it is expected that the pandemic increase the rate of new tracheostomies worldwide [9, 10]. In addition, the technique and the exposure of the medical staff to the aerosol during the procedure increases the rate of infection especially in resource-limited hospitals [11]. 

The concept of early tracheostomy (5-7 days) in patients requiring long-term mechanical ventilation has been supported by several lines of evidence in patients with traumatic brain injury (TBI) and ARDS [12, 13]. In COVID-19 patients, the concept has also been tested and the results are promising [14, 15]. Early tracheostomy is associated with increased patients’ mobility, decreased sedations, improved respiratory physiology and decreased length of ICU and hospital stay [15]. In addition, early tracheostomy decreases the rate of respiratory infections and used antibiotics in trauma patients [12, 13]. Recently, Williamson *et al*. [15] presented their experience with 29 early (7-10 days) percutaneous tracheostomy in patients with COVID-19. They demonstrated that early tracheostomy was associated with substantial decrease in sedation and vasopressor requirements [15]. According to these results, we can conclude that early tracheostomy may preserve the resources for further patients during the pandemic. 

The dilemma rises when two conditions are encountered simultaneously: the trauma and COVID-19. During the pandemic, many patients with trauma presenting to trauma centers had simultaneous COVID-19 pneumonia requiring treatment for both conditions. Several guidelines and protocols have developed for self-protection and management of co-existence of trauma and COVID-19 infection [16]. In those COVID-19 patients who experience the ARDS and suffer from traumatic injuries, the management is controversial and associated with poor outcome [16]. Thus, early weaning off the ventilator and decreasing sedation and mobilization of the patient is off clinical value and might decrease the mortality and morbidity of this patients. In addition, critically ill patients with COVID-19 infection and ARDS benefit from extracorporeal membrane oxygenation (ECMO) according to the recently published data from China [17]. Tracheostomy could assist the patients to benefit from ECMO. Thus, early tracheotomy and ECOM in selected patients should be considered to decrease the mortality and morbidity. 

In conclusion, based on the published evidence and our previous experience in trauma patients, we postulate that early tracheostomy could be associated with improved outcome in patients with COVID-19 and ADRS experiencing traumatic injuries. This is based on decreased need for sedation, and vasopressor, decreased rates of secondary infection and early mobilization along with get benefit of ECMO. However, the decision for early tracheostomy should be made based on the clinical scenario on a case‐by‐case basis. The procedure should be performed in accordance to the self-protection guidelines to maintain the safety of the healthcare team especially in resource limited hospital. 

## Conflict of Interest:

None declared.
